# RNA-seq data of *Oryza sativa* cultivar Kuku Belang under PEG treatment

**DOI:** 10.1016/j.dib.2017.07.043

**Published:** 2017-07-20

**Authors:** Bernadette Toni, Hossein Hosseini Monfared, Mohd Noor Mat Isa, Nurulhikma Md Isa, Ismanizan Ismail, Zamri Zainal

**Affiliations:** a Institute of Systems Biology, Universiti Kebangsaan Malaysia, UKM, 43600 Bangi, Selangor, Malaysia; b School of Bioscience and Biotechnology, Faculty of Science and Technology, Universiti Kebangsaan Malaysia, UKM, 43600 Bangi, Selangor, Malaysia; c Malaysia Genome Institute, Jalan Bangi, 43000 Kajang, Selangor, Malaysia

## Abstract

Drought stress is the main abiotic factor affecting rice production. Rain-fed upland rice which is grown on unbounded fields and totally dependent on rainfall for moisture is more prone to drought stress compared to rice from other ecosystems. However, upland rice has adapted to this limited water condition, thus are more drought tolerant than rice from other ecosystems. We performed the first transcriptome sequencing of drought tolerant indica upland rice cultivar Kuku Belang to identify differentially expressed genes related to drought tolerance mechanism. Raw reads for non-treated and PEG-treated *Oryza sativa* subspecies indica cv. Kuku Belang were deposited in the NCBI SRA database with accession number SRP074520 (https://www.ncbi.nlm.nih.gov/sra?term=SRP074520).

**Specification Table**

**Table:** 

Subject area	*Biology*
More specific subject area	*Molecular biology of stress response*
Type of data	*Transcriptome data*
How data was acquired	*Transcriptome of O. sativa cv. Kuku Belang was sequenced using Illumina HiSeq™ 2500 at Malaysia Genome Institute (MGI). Raw reads was processed and genome-guided assembly was performed using Tuxedo protocol which includes TopHat (*http://tophat.cbcb.umd.edu/*) and Cufflink (*http://cufflinks.cbcb.umd.edu*). Differentially expressed genes were identified using CuffDiff.*
Data format	*Raw sequence (fastq)*
Experimental factors	*Non-treated and PEG-treated seedlings*
Experimental features	*Two weeks old rice seedlings were immersed in 20% PEG-6000 to mimic drought stress. For control, seedlings were immersed in distilled water. Sampling was performed at multiple time points (6,12,18,48,72 and 96 h). Exact masses of total RNA extracted from rice seedlings treated with PEG for 6,12,18,48,72 and 96 h were pooled into one sample (treated sample). Similarly, exact masses of total RNA extracted from rice seedlings treated with distilled water for 6,12,18,48,72 and 96 h were pooled into one sample (non-treated sample). Treated and non-treated samples were sent for paired-end sequencing using Illumina platform.*
Data source location	*O. sativa cv. Kuku Belang was sown in the glass house at UKM Bangi, Selangor, Malaysia (2°55′14.5′′N 101°47′01.4′′E)*
Data accessibility	*Raw reads for non-treated and PEG-treated O. sativa subspecies indica cv. Kuku Belang were deposited in the NCBI SRA database with accession number SRP074520 (*https://www.ncbi.nlm.nih.gov/sra?term=SRP074520*).*

**Value of data**

• Upland rice which is better adapted to drought condition is more drought tolerant compared to lowland, irrigated or deep-water rice.
• Identification of genes responsible for drought tolerant traits of upland rice is therefore important for improvement of rice production under unfavorable conditions such as drought which is getting worse due to global climate change and diminishing water resources.
• Sequencing of drought tolerant upland indica rice cv. Kuku Belang and RNA-seq analysis of the transcriptome helps in identification of differentially expressed genes which are related to drought tolerance mechanism thus unraveling the underlying mechanism of drought tolerance in upland rice at molecular level.

## 1 Data

Transcriptome data of *Oryza sativa* subspecies indica cv. Kuku Belang were generated from the polyA-enriched cDNA libraries prepared from total RNA extracted from two weeks old seedlings treated with PEG (treated sample) and distilled water (non-treated sample). Short reads were filtered, processed, assembled and analysed as describe in the next section. Raw data for this project were deposited in the NCBI SRA database with accession number SRP074520 (https://www.ncbi.nlm.nih.gov/sra?term=SRP074520).

## 2 Experimental design, materials and methods

### 2.1 Plant materials and sample preparation

Seeds of *O. sativa* indica cv. Kuku Belang obtained from Malaysian Agricultural Research and Development Institute (MARDI), Seberang Prai were sterilised, germinated, and sown in glass house (2°55′14.5′′N 101°47′01.4′′E) with the temperature at 26/22 °C (day/night), 75/70% humidity, day length of 12 h, and light intensity of 700 µmol m^−^^2^ s^−2^. To mimic drought stress, two weeks old seedlings were treated with PEG by immersing its roots for 6,12, 18, 48, 72, and 96 h in 20% PEG-6000 solution whereas for non-treated samples, the roots were immersed in distilled water. Samples were collected at the designated time points and frozen in liquid nitrogen before being stored at −80 °C.

### 2.2 Total RNA extraction and quality control, library preparation and RNA-seq

Exact masses of total RNA extracted from rice seedlings treated with 20% PEG-6000 for 6,12,18,48,72 and 96 h were combined into one sample (treated sample). Similarly, exact masses of total RNA extracted from rice seedlings treated with distilled water for 6,12,18,48,72 and 96 h were combined into one sample (non-treated sample). Total RNA was extracted using TRIzol reagent as described by the manufacturer (Life Technologies). Total RNA purity was confirmed using Nanodrop 1000 (Thermo Fisher Scientific Inc., USA) whereas total RNA integrity was confirmed using 1% agarose gel electrophoresis. DNA contamination was removed using RNAse-free DNase kit as described by the manufacturer (Thermo Scientific). Both of treated and non-treated samples were sent for sequencing at Malaysian Genome Institute (MGI).

PolyA-enriched cDNA library was prepared using TruSeq Stranded Total RNA Sample Preparation with Ribo-Zero Plant kit as described by the manufacturer (Illumina). PEG-treated sample was indexed using TruSeq Adapter Index 14 whereas non-treated sample was indexed using TruSeq Adapter Index 7. Quality of cDNA library prepared were analysed using Agilent Technologies 2100 Bioanalyzer (Agilent Technologies, USA). Clustering was performed using cBot (version 1.4) and TruSeq PE Cluster v3 kit (Illumina). Paired-end sequencing of 101 bp was then performed using Illumina HiSeq™ 2500 and TruSeq SBS v3 kit (Illumina).

### 2.3 Assembly and RNA-seq analysis

**Fig. 1:**
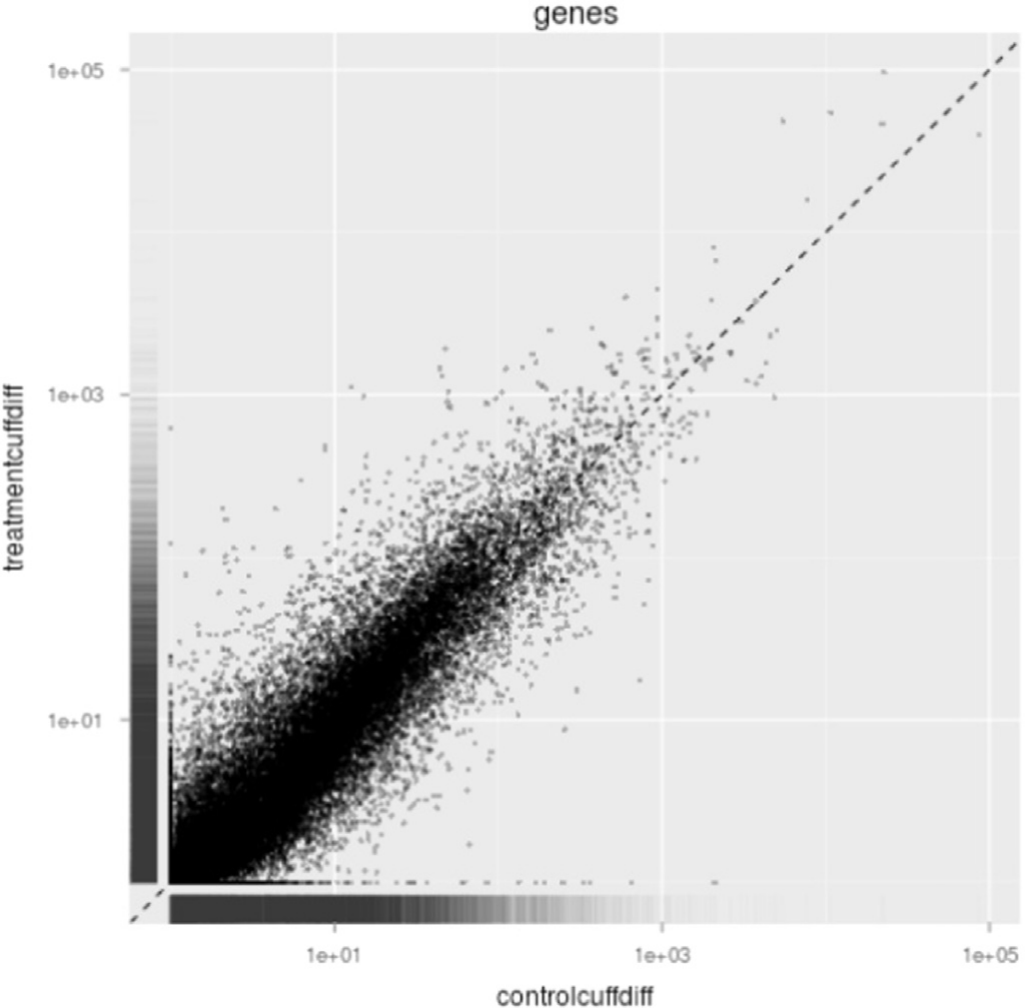
Scatter plot created from gene expression data (FPKM values) of PEG-treated and non-treated samples using CummeRbund showing distribution of genes with similar expression values which concentrates near the diagonally dotted straight lines and outliers which deviates from the diagonally dotted straight lines. FPKM, fragments per kilobase of transcript per million fragments mapped.

**Fig. 2:**
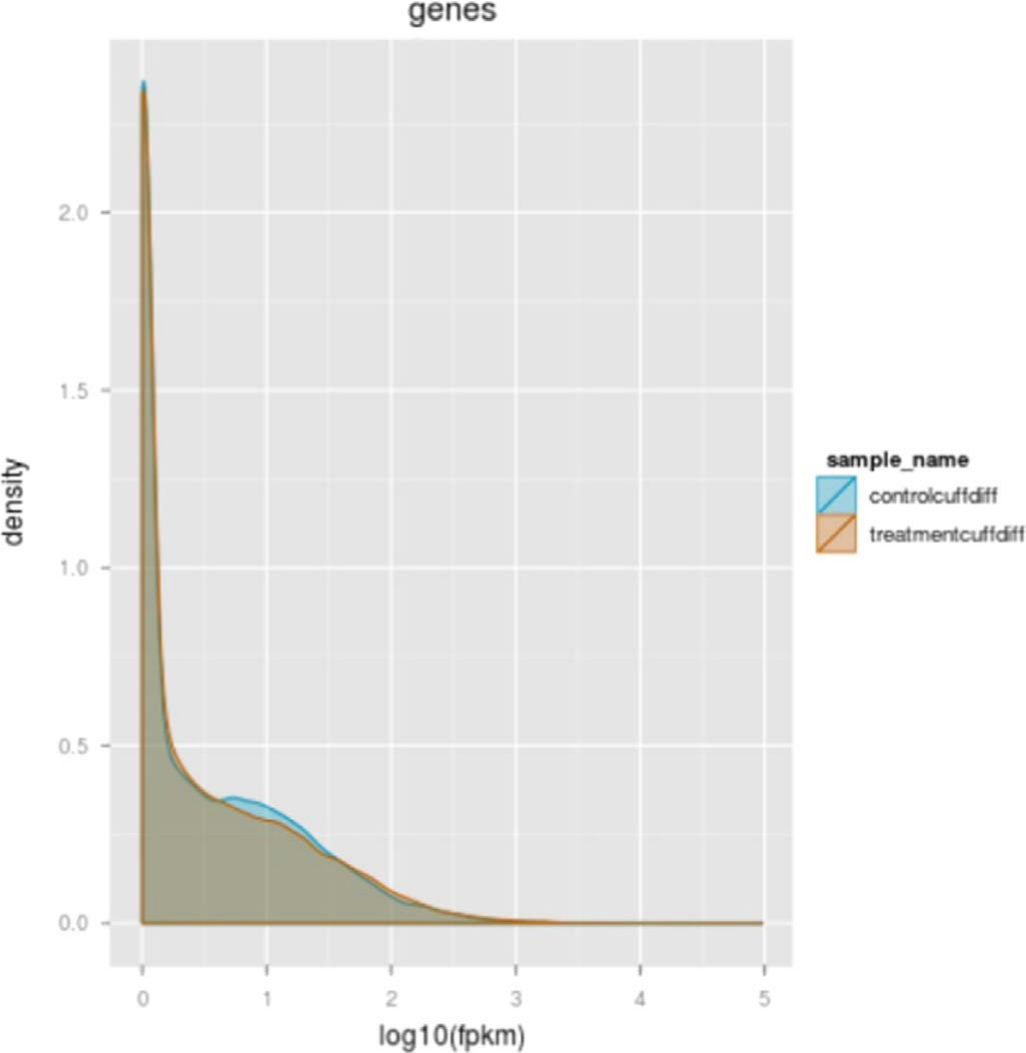
Density plot showing the distribution of RNA-seq read counts (FPKM) of PEG-treated (orange area) and non-treated (blue area) samples created using CummeRbund. Most genes in PEG-treated and non-treated samples has similar distribution of RNA-seq read counts (grey area).

**Fig. 3:**
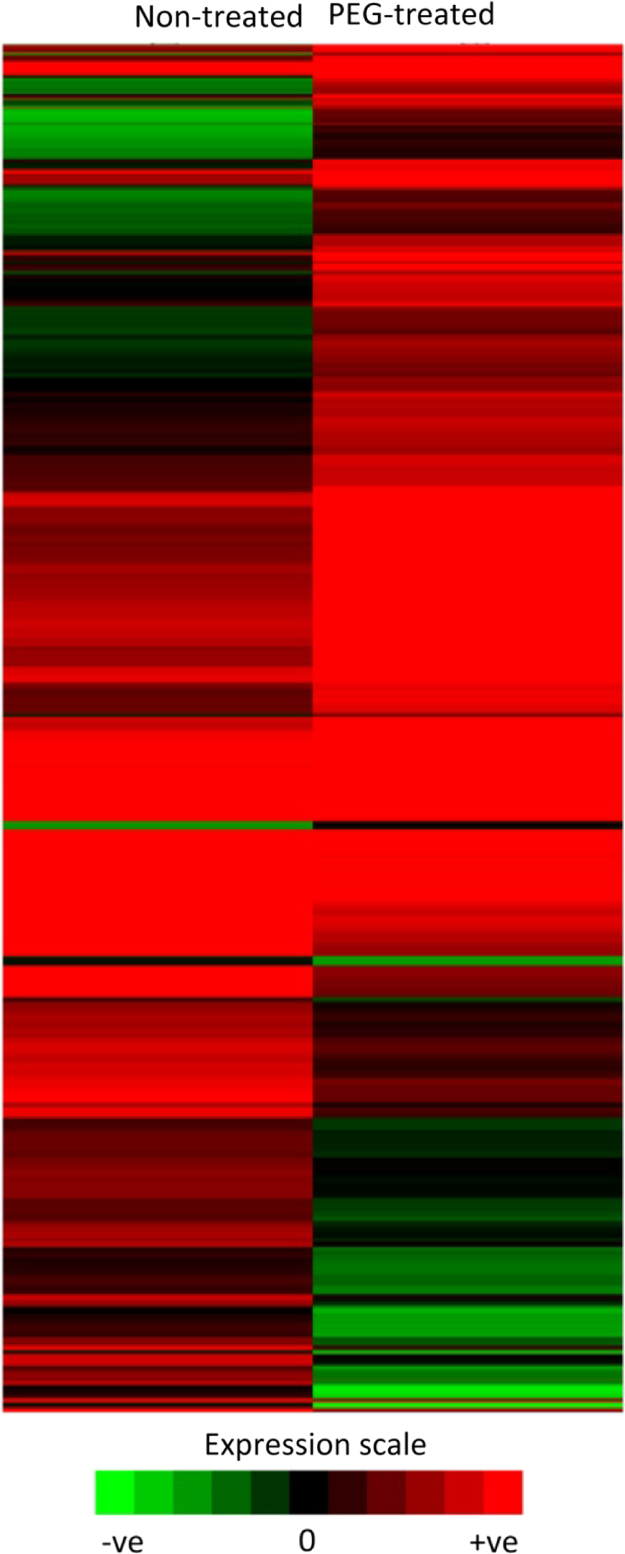
Heat map of differentially expressed genes in non-treated and PEG-treated samples created using Cluster and Treeview. Gene expression values used to create the heat map are the log_2_ FPKM of the differentially expressed genes in non-treated and treated samples.

High quality raw reads with Phred score ≥ 30 generated from sequencing of PEG-treated and non-treated samples were kept for assembly. Genome-guided assembly was performed using the Tuxedo [1] protocol whereby the high quality raw reads of both samples were mapped independently to the reference genome used which is the *O. sativa* subspecies indica genome ASM465v1.15 using TopHat (v2.0.4) [2]. The alignment files of both samples were then fed independently to Cufflink (v2.0.1) [3]. Next, the assembled transcripts from both samples were merged to produce final transcriptome assembly using Cuffmerge [4]. Cuffmerge [4] was also used to merge the final transcriptome assembly with the reference genome annotation. CuffDiff was used to quantify transcripts abundance (FPKM) in both samples and identify differentially expressed genes according to gene expression level and statistical significance test. Genes with log_2_ fold change ≥ 2, *p*-value ≤ 0.001 and *q*-value ≤ 0.05 were considered differentially expressed. Expression plots such as scatter plot (Fig. 1) and density plot (Fig. 2) were generated using CummeRbund (v2.0.0) [5]. Heatmap was generated using Cluster 3.0 [6] and Treeview (v1.1.6r4) [7] (Fig. 3). Table 1 shows the sequencing and RNA-seq statistics. Lists of differentially expressed genes were provided as Supplementary material.

**Table 1:** Sequencing and RNA-seq statistics of *O.sativa* indica cv. Kuku Belang.

Attributes	Value
*Raw reads*	
Total number (PEG-treated)	42,711,734
Total number (non-treated)	43,693,104
Total bases (PEG-treated)	4,313,885,134
Total bases (non-treated)	4,413,003,504
Q30 (%) (PEG-treated)	97.59
Q30 (%) (non-treated)	97.43
*Filtered reads*	
Total number (PEG-treated)	42,107,560
Total number (non-treated)	41,916,310
Total bases (PEG-treated)	3,802,599,685
Total bases (non-treated)	3,747,719,542
Q30 (%) (PEG-treated)	99.11
Q30 (%) (non-treated)	99.03
*Mapped reads*	
Total number (PEG-treated)	34,537,364
Total number (non-treated)	33,050,552
Exon (PEG-treated)	21,557,422
Exon (non-treated)	20,164,201
Exon-exon junction (PEG-treated)	11,778,928
Exon-exon junction (non-treated)	11,573,604
Intron (PEG-treated)	1,201,014
Intron (non-treated)	1,312,746
*Differential gene expression analysis*	
Differentially expressed genes	541
Up-regulated genes	365
Down-regulated genes	176
